# Crosstalk regulation among group 2- Sigma factors in Synechocystis PCC6803

**DOI:** 10.1186/1471-2180-5-18

**Published:** 2005-04-22

**Authors:** Sylvain Lemeille, Johannes Geiselmann, Amel Latifi

**Affiliations:** 1 Laboratoire Adaptation et Pathogénie des Microorganismes, Université Joseph Fourier Bâtiment Jean Roget – Faculté Médecine-Pharmacie. Domaine de la Merci. 38700 La Tronche, France; 2 Laboratoire de chimie bactérienne, IBSM-CNRS, 31 chemin Joseph Aiguier. 13402 Marseille cedex 20, France

## Abstract

**Background:**

The cyanobacterium *Synechocystis *PCC6803 contains one group 1 (*sigA*) and four group 2 (*sigB*, *sigC*, *sigD *and *sigE*) sigma factors. The activity of these multiple sigma factors determines the transcriptional program of this bacterium. We wanted to study the role of the group 2 sigma factors in *Synechocystis*. We have therefore constructed mutants of each of the group 2 sigma factors and investigated their crosstalk.

**Results:**

We used quantitative RT-PCR analysis to measure the relative abundance of the *sig *mRNAs in the four sigma mutants. Our data indicate that a network of mutual transcriptional regulation links the expression of the sigma genes. Accordingly, an environmental stress acting on only one of the sigma factors will indirectly modify the expression of most of the other sigma factors. This was confirmed by the transcriptional analysis of the *sig *mRNAs as a function of nitrogen starvation.

**Conclusion:**

Taken together, our observations suggest that the crosstalk regulation between all group 1 and group 2 genes could be important for the adaptation of the bacterium to different environmental and physiological conditions.

## Background

Bacterial sigma subunits of RNA polymerase are global regulators of gene expression, conferring specificity to the recognition of promoters by the core enzyme. Two broad families of sigma factors have been identified: the σ^70 ^type, and the σ^54 ^type factors [1]. The σ^54 ^family regulates a variety of genes such as those involved in chemotaxis, synthesis of structural components of flagella and enzymes involved in the response to nitrogen starvation [2]. The σ^70 ^family is subdivided into three groups [1]. Group 1 comprises the primary sigma factors that control the transcription of housekeeping genes, and these sigma factors are therefore essential for cell viability. Groups 2 and 3 include the so-called alternative sigma factors that coordinate the regulation of gene expression in bacteria on a global level. They direct the transcription of a specific genetic program that allows bacteria to cope with particular environmental changes and stress conditions. Group 2 sigma factors are similar in sequence to primary sigma factors and include proteins such as the stationary-phase-specific sigma factor, RpoS [3]. Group 3 sigma factors show less sequence similarity with those of group 1 and include proteins required for the heat shock response [4] and motility [5]. The inactivation of a gene encoding a particular groups 2 or group 3 sigma factor usually produces growth defects or other phenotypes under specific physiological or environmental conditions. An *E. coli rpoS *mutant, e.g., has a pleitropic phenotype: it shows a loss of viability in stationary phase and a decreased resistance to some stresses such as the osmotic stress [6]. In *Synechocystis *PCC6803, inactivation of the *sigF *gene, encoding a group 3 sigma factor, leads to the loss of motility and pilus formation [7]. In *Synechococcus *four mutants of *rpoD *genes show defects in the circadian expression of the *psbAI *gene, encoding the protein D1 of the photosystem II reaction center [8].

The unicellular cyanobacterium *Synechocystis *sp. strain PCC6803 possesses one group 1 sigma factor, *sigA *(slr0653), four group 3 sigma factors (sll 0687, sll0856, slr1545, slr1564) and four group 2 sigma factors, *sigB *to *sigE *(sll0306, sll0184, sll2012, sll1689) [9]. SigE is involved in the response to nitrogen stress [10] and a contribution of the SigB/SigD factors to the dark/light adaptation has been reported recently [11]. The synthesis of the other alternative sigma factors is also modulated in response to particular stresses [12-14].

In order to assess the role of alternative sigma factors in *Synechocystis*, we have chosen to study the group 2 sigma factors. We have analyzed the transcription of all members of this family of *sig *genes as well as the transcription of the group 1 sigma factor in *Synechocystis *PCC6803 wild type strain and mutants lacking the group 2 sigma genes. Based on our results we suggest that these sigma factors are linked by a network of mutual regulation that could allow them to act in concert in the global transcriptional control of this bacterium.

## Results and discussion

### Construction and growth of sigma mutants

**Figure 1:**
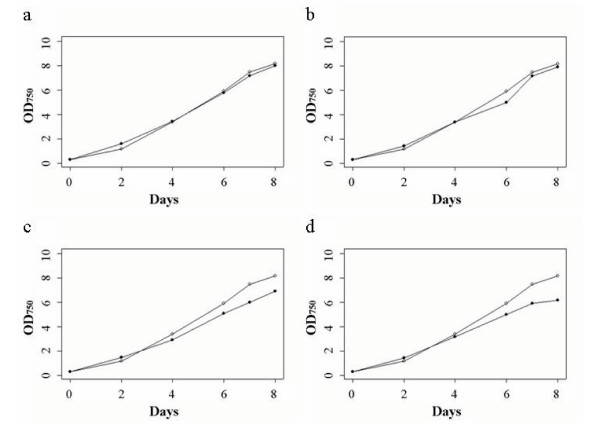
Growth of the *sig *mutants (solid circle) compared to the wild type strain (open circle) under normal conditions. a: *sigB *mutant, b: *sigC *mutant, c: *sigD *mutant, d: *sigE *mutant. Wild type and mutant strains were grown in Bg11 medium. Growth was monitored by measuring the optical density at 750 nm (A_750_).

We have constructed null mutant strains lacking either *sigB*, *sigC*, *sigD*, or *sigE *gene as described in Materials and Methods. These four mutants segregated completely (data not shown). We were not able to inactivate the *sigA *gene, suggesting that it is necessary for the viability of the organism. The growth of the *sigB*, *sigC*, *sigD*, or *sigE *mutants compared to the wild type strain was examined under normal growth conditions. The results of Figure 1 indicate that all four mutants have a similar growth rate as the wild type strain during the exponential phase, showing that these sigma factors are dispensable for growth under our culture conditions and confirming their classification as group 2 sigma factors.

### Expression of the group 2 sigma genes during normal growth

**Figure 2:**
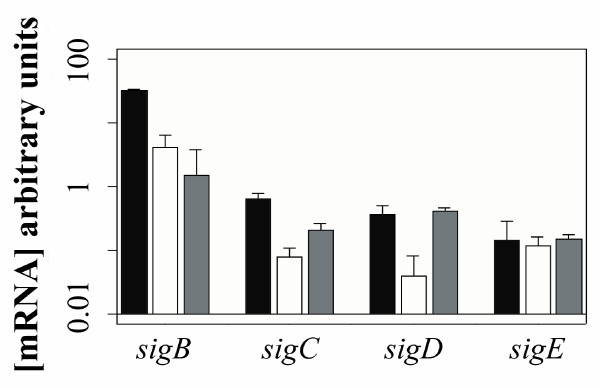
Trascription of *sig *genes during normal growth. Measurement of relative concentrations of *sigB*, *sigC*, *sigD *and *sigE *mRNAs in *Synechocystis *by quantitative RT-PCR during three stages of normal growth: mid log phase in black (A_750 _= 1); early stationary phase in white (A_750 _= 8); and late stationary phase in grey (A_750 _= 12.6). Each sample was measured in triplicate and the standard deviation is indicated by error bars. Values were normalized to the *rpoA *transcript.

Recent studies have shown that all four group 2 sigma genes are expressed in normally growing cells [12]. We wanted to assess if their transcription changes in response to a gradual physiological modification of the internal and external environment of the bacterium. Our analysis is based on quantitative RT-PCR. We quantified the amount of the *sigB*, *sigC*, *sigD *and *sigE *transcripts during three stages of growth: mid log, early stationary phase and late stationary phase. The level of the transcripts was normalized to the level of *rpoA *transcription as described in Material and Methods. The results presented in Figure 2 show that all four group 2 sigma factors are transcribed in all growth phases. However, the relative abundance of the different sigma factors changes all along the growth of the culture. The *sigB *transcript was maximum during exponential phase, and decreased as cells grew into stationary phase. *sigC *and *sigD *transcription decreased upon entry into stationary phase and increased as cells were adapted to this stage of growth, *sigE *had the weakest variation. Imamura et al. [12] have shown that the protein concentrations of three (SigC, SigD, SigE) sigma factors vary in a similar manner as in our transcriptional analysis, suggesting that, at least for these factors, RNA levels correlate well with protein amounts.

These expression profiles show that the transcript levels of at least three of these sigma factors depend on the cell density (A_750 _value) of the culture and suggest that they could be important for the global physiology of this bacterium at all stages of growth.

All sigma factors are expressed in many environmental conditions tested, such as iron and sulfur starvations, and heat and osmotic shocks (data not shown). Similar results were previously obtained in *Synechococcus elongatus *PCC7942 [15] where all sigma genes were found to be active under many growth conditions. Recently Imamura et al. [12] measured the concentration of all five sigma factors during normal growth of *Synechocystis *PCC6803. They found the amounts of these proteins to vary between 1 and 10 fmoles/μg of total protein. These data suggest that all five factors are important for the cellular physiology of *Synechocystis *PCC6803 under standard conditions. This conclusion is somewhat surprising since four of the five *sig *genes can be mutated, indicating that neither of them is essential for viability under these conditions. One possible way to reconcile these two divergent observations would be to suppose that the function of the different sigma factors is redundant. According to this model, most genes would be transcribed by more than one sigma factor. Indeed, the sigma genes themselves are transcribed from multiple promoters [12]. Furthermore, it is even possible that the different sigma factors recognize the same promoter. This hypothesis is supported by data obtained from *in vitro *transcription experiments in which all three different sigma factors (RpoD1, RpoD3, RpoD4) could initiate the transcription of the *rrnA*, *cpcB1A1 *and P1a promoters of *Synechoccocus *sp. strain PCC7942 [15]. This specificity crosstalk among sigma factors is also revealed by an *in vivo *analysis of *psbAI *promoter activities in *S. elongatus *where the principal sigma factor, as well as each group 2 sigma factor, all recognize the *psbA1 *promoter of this bacterium [16].

### Transcription of *sig *genes in σ mutants

**Figure 3:**
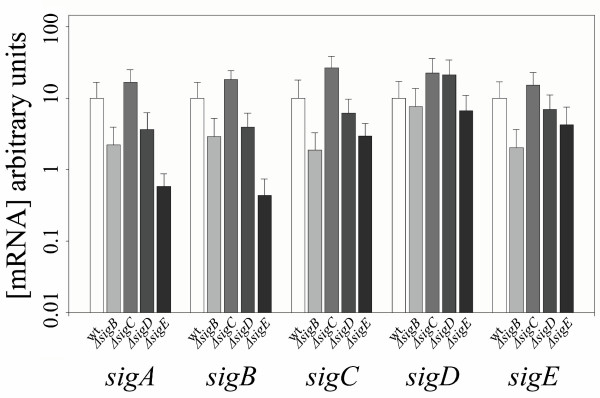
Transcription of *sig *genes in sigma mutants. *sigABCDE *genes were quantified in the wild type and the four sigma mutants (*sigB*, *sigC*, *sigD *and *sigE*). The gene quantified is indicated below each set of measurements. Within each set, the phenotype is indicated below each bar of the graph. All measurements were carried out under identical culture conditions for the wt and mutants strains. Values were normalized to the *rpoA *transcript. The expression of each gene in the wt strain was set equal to 10 (in arbitrary units). The reported values represent the average of 6 measurements obtained from two separate RNA preparations.

Since the *sig *genes are transcribed by RNA polymerase holoenzyme, they necessarily regulate each other's transcription. In other systems it is well documented that the transcription of alternative sigma factor genes is controlled by other σ factors [17-19]. In order to investigate this regulatory network, we measured the transcription of each of the five *sig *genes in all four σ mutants during exponential growth. Our method can detect transcripts of all *sig *genes in all of the mutants because the cDNA synthetized during the RT-PCR used primers that anneal upstream of the inactivating chloramphenicol cassette. The results shown in Figure 3 confirm the existence of complex regulatory connections between the different sigma factors and suggest a highly interconnected network: (i) mutation in the *sigB *gene leads to a 6-fold decrease of *sigA*, *sigC *and *sigE *genes (Figure 3), (ii) in the *sigD *mutant, transcription of the *sigA*, *sigB*, *sigC *and *sigE *genes decreased about 3 to 4-fold (Figure 3), (iii) sigE mutation leads to a strong decrease (about 20-fold) of the transcription of the *sigA *and *sigB *genes and to a 5-to 3-fold decrease of the expression of the *sigC *and *sigD *genes (Figure 3), (iii) mutation in the *sigC *gene does not negatively affect the transcription of any of the 4 sigma genes tested (Figure 3).

SigE seems to be a particularly important sigma factor because it controls the expression of three other *sig *genes. Mutation of the *sigE *gene had the strongest effects among all mutants inactivating sigma genes, affecting particularly the housekeeping gene *sigA *and *sigB*. The role of the housekeeping sigma factor, SigA, remains less well defined. Since inactivation of this gene is lethal, we will have to investigate its role using conditional mutants or biochemical methods.

By quantifying the sigma transcripts in different sigma mutants we have shown that the transcription of the *sig *genes is controlled by a network of mutual connections between the sigmas. Previous studies in related organisms had also shown a mutual transcriptional regulation of sigma factors. In *Synechococcus *PCC7942, the *rpoD1 *gene is transcribed by RpoD3 and RpoD4 factors [15] and SigC factor has a negative effect on SigB expression [11]. In *Borrelia burgdorferi*, for example, RpoN regulates the expression of *rpoS *[18].

### Sigma factors transcription under nitrogen starvation

**Figure 4:**
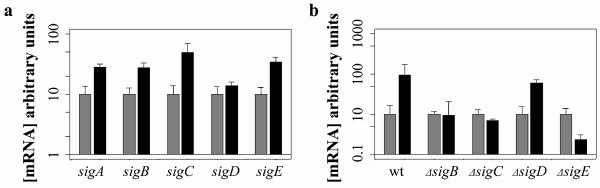
a: Levels of sigma factor mRNAs during nitrogen starvation. Cells were grown under normal (white columns) or nitrogen starved (grey columns) conditions. *sig *mRNAs were quantified using quantitative RT-PCR. Each sample was measured in triplicate and the standard deviation is indicated by error bars. Values were normalized to the *rpoA *transcript. The expression of each gene in non starved cells was set equal to 10 (in arbitrary units). b: Effect of sigma mutations on the transcription of *glnN *gene during nitrogen starvation. mRNAs were quantified using quantitative RT-PCR. Each sample was measured in triplicate and the standard deviation is indicated by error bars. Values were normalized to the *rpoA *transcript. The expression of *glnN *gene in non starved cells (white columns) was set equal to 10 (in arbitrary units). The grey bars represented the expression of *glnN *gene in nitrogen starved cells.

According to the network of mutual transcriptional regulation of the sigma factors, we speculated that an environmental stress acting on only one of the sigma factors will indirectly modify the expression of most of the other sigma factors. We tested this hypothesis by analyzing the transcription profiles of the group 1 sigma gene and the four group 2 sigma factors under nitrogen starvation. We measured the mRNA concentration of the group 1 sigma gene and the four group 2 genes by quantitative RT-PCR under nitrogen stravation. As shown in Figure 4a, *sigE *expression was induced, as expected, about 5-fold with respect to the reference gene *rpoA*. The transcription of the other four sigma genes was also induced (about 2- to 6-fold). Nitrogen starvation does not only lead to the overexpression of the *sigE *gene but rather provoked a readjustment of the relative abundance of the sigma factors. This global change of the expression of all sigma genes in response to this particular environmental condition agrees with our hypothesis that the cross-talk regulation among the sigmas could lead to the transmission of one particular signal to all of them. The transcription of a structural gene involved in this stress should be affected by more than one sigma factor. *glnN*, encoding the GSIII glutamine synthase, known to be highly expressed under conditions of nitrogen deficiency [10,17] was chosen as a target gene for the nitrogen starvation. We have analyzed its transcription in the wild-type and mutant strains after one week of nitrogen starvation. Induction of *glnN *transcription was observed in the wild type strain. This induction was abolished when SigB, SigC or SigE factors were inactivated (Figure 4b). These results clearly demonstrated that more than one sigma factor affects the transcription of the *glnN *gene.

Our analysis is based on measuring the first level of control of *sig *genes expression. In other systems, post-transcriptional modifications of sigma factors can occur and may not correlate with transcriptional profiles. The recent data of Imamura et al [12] suggest however that the intracellular concentration of the sigma factors correlates well with the transcriptional control of these sigma factors (with the possible exception of SigB). The same authors demonstrated that a negative effect of SigC on *sigB *transcription correlates with the reduction of SigB protein levels in this mutant [12]. These data suggest that transcriptional regulation of *sig *genes is not drastically modified by post-transcriptional control. This hypothesis is actually under investigation in our laboratory.

## Conclusion

**Figure 5:**
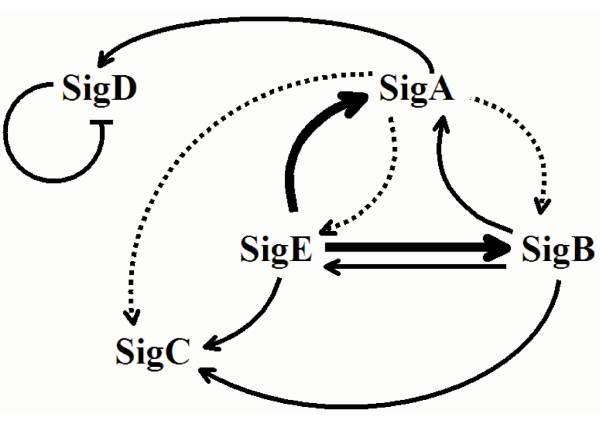
Schematic network of transcriptional interactions between group 2 sigma genes transcription in *Synechocystis*: The thickness of the arrows is proportional to the effect of a given mutation on the transcription of the sigma gene to which the arrow points.

*Synechocystis *PCC6803 possesses multiple sigma factors and its transcriptional program is largely determined by the activity of these multiple sigma factors. Our study has explored the relationship among all members of the group 2 sigma factors and their connection with the housekeeping sigma factor. We have shown that the transcription of the *sig *genes is controlled by a network of mutual connections. The strongest effects are compiled in Figure 5, where the thickness of the arrows is proportional to the effect of a given mutation on the expression of the sigma genes. For example, mutating the *sigE *gene results in the reduced expression of four sigma genes, with a more pronounced effect on the *sigA *and *sigB *genes. A mutation in the *sigB *gene results in a small reduction of the transcription of the *sigA*, *sigE *and *sigC *genes. Expression of the group 1 sigma gene (*sigA*) is affected by mutations of three group 2 sigma genes. Our study has explored the relationship among all members of one family of sigma factors in eubacteria. We assume that their mutual connections are part of a more extended regulation network. In fact, it is tempting to speculate that all sigma factors in a cell control each other. A possible connection to group 3 sigma factors, as well as the relationships among sigma factors and sensor and regulatory proteins in the cell remain to be elucidated.

## Methods

### Culture and growth conditions

*Synechocystis *sp. strain PCC6803 was obtained from the Pasteur culture collection. Wild-type and mutant strains were grown at 30°C with continuous illumination at approximately 20 μE m^-2 ^s^-1^, with 3% CO_2 _in air, in BG11 medium [20], buffered with 5 mM Hepes-KOH, pH8. Growth was monitored by measuring the optical density at 750 nm (A_750_).

For nitrogen starvation, BG11 medium lacking the nitrogen source (NaNO_3_) was buffered with 20 mM *N*-tris(hydroxymethyl)methyl-2-aminoethanosulfonic acid (TES) buffer, pH 7.5. Strains used in this condition were grown to an A_750 _= 1, transferred to the nitrogen-depleted medium and incubated for one week. All cyanobacterial strains were grown on BG11 plates containing 1.5% Difco Bacto Agar. When needed, chloramphenicol was added to a concentration of 10 μg/ml. Growth rates of mutants were compared to a *Synechocystis *strain carrying the same antibiotic resistance cassette inserted into an inessential gene, *ureA*. We call this strain the wild-type for our experiments.

### DNA manipulation and RNA isolation

Molecular techniques were performed according to standard procedures [21]. *Synechocystis *genomic DNA was prepared according to the method of Tandeau de Marsac et al. [22]. RNA was extracted from pelleted cells, broken by freezing in liquid nitrogen, and using the RNeasy kit (Qiagen) according to the manufacturer's specifications. Chromosomal DNA was removed by treating RNA preparations with 1 μl of DNAse (at 2U/μl) (Ambion) for 1 hour at 37°C. The concentration of RNA was determined spectrophotometrically.

### Gene inactivation

The *sigB *(*sll0306*), *sigC *(*sll0184*), *sigD *(*sll2012*) and *sigE *(*sll1689*) genes [9] were cloned using the TOPO-TA cloning kit (Invitrogene) and the following primers: *sigB*-1 and *sigB*-2, *sigC*-1 and *sigC*-2, *sigD*-1 and *sigD-2*, *sigE*-1 and *sigE*-2.

These genes were then subcloned into pBluescript SK- plasmid (Stratagene) between the *ApaI *and *SpeI *sites. A chloramphenicol cassette was inserted at the unique site *Bgl*II in *sigC*, *Sma*I in *sigB*, and *BamH*I both in *sigE *and *sigD*. The cassette was obtained from the pACYC184 plasmid [23] by PCR amplification. The primers used were *cat-1 *and *cat-2*, they add *BamH*I and *SmaI *restriction sites at each end of the amplified sequence.

The SK- vector derivatives containing each interrupted gene were used to transform *Synechocystis*. Chloramphenicol-resistant transformants that were also ampicillin sensitive were selected and subsequently screened for replacement of the wild-type gene allele with the corresponding mutant. Genomic DNA isolated from individual Cm^R ^transformants was verified by PCR.

### Reverse transcription

For each reaction, 1 μl of antisens primer mix at 2.5 μM of each of the primers and 200 ng of total RNA were denaturated at 95°C and chilled quickly on ice. A mix consisting of 4 μl of 5x buffer (250 mM Tris-HCl pH 8.3, 375 mM KCl, 15 mM MgCl_2_, 50 mM DTT), 0.5 μl of RNase Inhibitor (40U/μl), 1 μl of 5 mM dNTP and 1 μl of MMLV reverse transcriptase enzyme (200U/μl) was added in a total volume of 20 μl, followed by one hour of incubation at 37°C. The composition of the primer mix used in each reverse transcription reaction was dependent on the genes analyzed in the quantitative PCR experiments (for example when *rpoA *and *glnN *genes were quantified, the reverse transcription reaction used *rpoA*-RT and *glnN*-RT primers). The primers used were: *rpoA*-RT; *sigB*, *C*-RT; *sigA*, *D*, *E*-RT; *glnN*-RT.

The sequences of all the primers used in this study are listed in table 1.

### Real-time quantitative PCR

PCR conditions were identical for all reactions. The 25 μl-reaction mixture consisted of 1x master mix buffer (Eurogentec), 0.75 μl of SYBR Green I Dye (Eurogentec), and 1 μl of each primer (2 μM). 5 μl aliquots of the diluted reverse transcription reaction were used as template. PCR amplifications (2' at 50°- 10' at 95° – 40 X [15 '' at 95° – 1' at 60°]) were carried out in a Gen Amp 5700 sequence detection system (Applied Biosystems). The forward (F) and reverse (R) primers used in these PCR reactions were designed using the Primer Express software (Applied Biosystems). For each RT-PCR reaction, the efficiency of the DNAse treatment was verified by an identical parallel PCR reaction, but omitting reverse transcription. Only DNA-free RNAs were used in our experiments. All PCR primers anneal to their target at temperatures comprised between 58 and 60°C and amplify 200-pb fragments internal to the coding sequence of the relevant gene. The primers used in the quantitative-PCR experiments are listed in table 1.

### Quantitative analysis of the sample

Real-time PCR quantitatively detects the concentration of double-stranded PCR products by monitoring the fluorescence of SYBR Green I Dye, which selectively binds to double-stranded DNA. All measurements were carried out in triplicates. The analysis method was based on the threshold cycle value (C_T_) that indicates the fractional cycle number at which the amount of amplified target reaches a fixed threshold. The standard deviations of our measurements are indicated by the error bars in the figures. Since the amount of *rpoA *transcript was practically constant in all the conditions tested in this study (table 2) we report the quantities of the other cDNAs with respect to the concentration of the *rpoA *transcript. We calculate the difference (ΔC_T_) between the mean C_T _of this sample and the mean C_T _value of the *rpoA *mRNA for that sample. The relative concentration of the mRNA species is then calculated as 2C^-ΔCT^.

## Authors' contributions

S L carried out all of the RT-PCR experiments and assembled the figures.

J G participated in the analysis of the data and the writing of the manuscript.

A L conceived the study, constructed the mutants and helped in writing the manuscript.

## Figures and Tables

**Table 1:** List of primers used in this study. All sequences are written from 5' to 3'.

Primer	Sequence
*sigB*-1	CGGAATTCTTGGGTATCTTTTTAGC
*sigB*-2	CCGAAGCTTGGGCAACTAACTGGC
*sigC*-1	CGGAATTCAAAGCCTGCCATCGGCC
*sigC*-2	CCGAAGCTTGTGGCCTAACCCAAATTTTC
*sigD*-1	CGGAATTCGAGTATGTGCTTCACAA
*sigD*-2	CCGAAGCTTCTTTTCTTTAGCTAGCT
*sigE*-1	CGGAATTCTTTGGAAAATCAATGACA
*sigE*-2	CCGAAGCTTGGTATCTATAACCAACC
*cat-1*	CGGGATCCCGCCCGGGAATTACGCCCCGCCC
*cat-2*	CGGGATCCCGCCCGGGCAGGAGCTAAGGAAGCTA
*rpoA*-RT	TAACCTA
*sigB*, *C*-RT	TAACCTT
*sigA*, *D*, *E*-RT	TAACCCT
*glnN*-RT	CCATCCGTC
*sigA *forward	TGGAGTTGGAAACCGCC
*sigA *reverse	GACTGCACCATTTTGTCTTTGG
*sigB *forward	AGAAATGGCCCGCTATCCC
*sigB *reverse	GCCCGCAGTTGATAAAGCC
*sigC *forward	TGGAGTTGGAAACCGCC
*sigC *reverse	GACTGCACCATTTTGTCTTTGG
*sigD *forward	GATGGCCCTGCTGGAGC
*sigD *reverse	TTGCGCTTCTGATATTTCTTGG
*sigE *forward	CGGGCCGCAGAATCC
*sigE *reverse	CCAACTCCGCCCATCG
*rpoA *forward	GAGTTCGCCACTATTCTAGGCG
*rpoA *reverse	TTAGGATCAATAACCTCCACCTC
*glnN *forward	GATTTAACCAAGGACGCTGGC
*glnN *reverse	CAAAACGGTTACCAGTGAAGGC

**Table 2:** *rpoA *C_t _values for the different growth conditions tested in this study. The reported values represent the average of 6 measurements obtained from two separate RNA preparations.

Culture condition or genetic background	rpoA C_t _value per 20 ng total RNA
Normal	28.5 ± 1.03
Nitrogen starvation	29 ± 0.27
	
Exponential phase	29.44 ± 0.03
Early stationary phase	28.60 ± 0.52
Late stationary phase	28.15 ± 0.04
	
Wild type strain	30 ± 0.91
*sigB *mutant	30.29 ± 0.25
*sigC *mutant	29.66 ± 1.06
*sigD *mutant	29.76 ± 0.8
*sigE *mutant	29.85 ± 0.69
